# A multicenter analysis of the clinical microbiology and antimicrobial usage in hospitalized patients in the US with or without COVID-19

**DOI:** 10.1186/s12879-021-05877-3

**Published:** 2021-02-27

**Authors:** Laura Puzniak, Lyn Finelli, Kalvin C. Yu, Karri A. Bauer, Pamela Moise, Carisa De Anda, Latha Vankeepuram, Aryana Sepassi, Vikas Gupta

**Affiliations:** 1 Merck & Co., Inc., Kenilworth, NJ USA; 2 Becton, Dickinson and Company, Franklin Lakes, NJ USA

**Keywords:** SARS-CoV-2, COVID-19, Epidemiology, Antibiotics, Pathogens, Coinfection

## Abstract

**Background:**

Past respiratory viral epidemics suggest that bacterial infections impact clinical outcomes. There is minimal information on potential co-pathogens in patients with coronavirus disease-2019 (COVID-19) in the US. We analyzed pathogens, antimicrobial use, and healthcare utilization in hospitalized US patients with and without severe acute respiratory syndrome-coronavirus-2 (SARS-CoV-2).

**Methods:**

This multicenter retrospective study included patients with > 1 day of inpatient admission and discharge/death between March 1 and May 31, 2020 at 241 US acute care hospitals in the BD Insights Research Database. We assessed microbiological testing data, antimicrobial utilization in admitted patients with ≥24 h of antimicrobial therapy, and length of stay (LOS).

**Results:**

A total of 141,621 patients were tested for SARS-CoV-2 (17,003 [12.0%] positive) and 449,339 patients were not tested. Most (> 90%) patients tested for SARS-CoV-2 had additional microbiologic testing performed compared with 41.9% of SARS-CoV-2-untested patients. Non-SARS-CoV-2 pathogen rates were 20.9% for SARS-CoV-2-positive patients compared with 21.3 and 27.9% for SARS-CoV-2-negative and −untested patients, respectively. Gram-negative bacteria were the most common pathogens (45.5, 44.1, and 43.5% for SARS-CoV-2-positive, −negative, and −untested patients). SARS-CoV-2-positive patients had higher rates of hospital-onset (versus admission-onset) non-SARS-CoV-2 pathogens compared with SARS-CoV-2-negative or −untested patients (42.4, 22.2, and 19.5%, respectively), more antimicrobial usage (68.0, 45.2, and 25.1% of patients), and longer hospital LOS (mean [standard deviation (SD)] of 8.6 [11.4], 5.1 [8.9], and 4.2 [8.0] days) and intensive care unit (ICU) LOS (mean [SD] of 7.8 [8.5], 3.6 [6.2], and 3.6 [5.9] days). For all groups, the presence of a non-SARS-CoV-2 pathogen was associated with increased hospital LOS (mean [SD] days for patients with versus without a non-SARS-CoV-2 pathogen: 13.7 [15.7] vs 7.3 [9.6] days for SARS-CoV-2-positive patients, 8.2 [11.5] vs 4.3 [7.9] days for SARS-CoV-2-negative patients, and 7.1 [11.0] vs 3.9 [7.4] days for SARS-CoV-2-untested patients).

**Conclusions:**

Despite similar rates of non-SARS-CoV-2 pathogens in SARS-CoV-2-positive, −negative, and −untested patients, SARS-CoV-2 was associated with higher rates of hospital-onset infections, greater antimicrobial usage, and extended hospital and ICU LOS. This finding highlights the heavy burden of the COVID-19 pandemic on healthcare systems and suggests possible opportunities for diagnostic and antimicrobial stewardship.

**Supplementary Information:**

The online version contains supplementary material available at 10.1186/s12879-021-05877-3.

## Background

Coronavirus disease 2019 (COVID-19), which is caused by severe acute respiratory syndrome coronavirus 2 (SARS-CoV-2), emerged in Wuhan, China in December of 2019 and became a pandemic. Past respiratory virus epidemics suggest an increased risk for bacterial, fungal, or other viral coinfections or superinfections due to biological factors, underlying conditions, and healthcare exposures [1, 2]. Varying coinfection and superinfection rates have been reported in patients with COVID-19, ranging from 5 to 33% depending on methods, populations, and geographic location (reviewed by Clancy and Nguyen [3] and Rawson et al. [4]). Overall, there is currently limited and often divergent information available on the frequency and microbiology of coinfections and superinfections in patients with COVID-19 [5]. Differences in the methods used for testing criteria, obtaining samples (particularly for nasopharyngeal sources), and storing specimens complicates data comparison and interpretation.

There are also limited data on antimicrobial usage in COVID-19 patients; available studies suggest that the use of broad-spectrum antibiotics is widespread in this population [4]. Such treatment patterns have potential implications for development of antibiotic resistance in both hospital and community settings [6], increased risk for other infections, such as *Clostridium difficile*, and potential adverse events and toxicity. More complete data on pathogen rates and antimicrobial usage are needed to support appropriate clinical management and antimicrobial stewardship policies during the pandemic [3, 4].

In this study, we used data from a large US hospital database to analyze and compare pathogen rates, antimicrobial utilization patterns, and health outcomes among SARS-CoV-2-positive, SARS-CoV-2-negative, and SARS-CoV-2-untested hospitalized adult patients.

## Methods

### Study design

We conducted a multi-center, retrospective cohort analysis of data from 241 US medical facilities. Reporting institutions were part of the BD Insights Research Database (Becton, Dickinson and Company, Franklin Lakes, NJ), which includes both small and large hospitals and medical care facilities in urban and rural areas throughout the US (BD Insights Research Database [Becton, Dickinson & Company, Franklin Lakes, NJ]) [7–9]. More information on these facilities can be found in the Results section and in Supplementary Table 1 (see Additional file 1). Eligible admissions were hospitalized patients with > 1-day inpatient admission and a record of discharge or death between March 1, 2020 and May 31, 2020. For purposes of comparison, patients were classified into 3 groups: (1) tested and positive for SARS-CoV-2; (2) tested and negative for SARS-CoV-2; and (3) not tested for SARS-CoV-2 (referred to as “untested”).

All microbiology results were based on local testing performed by individual microbiology labs in the cohort of hospitals in the BD Insights Research Database. SARS-CoV-2 status was determined by in-hospital or ambulatory PCR or antigen assays performed within 7 days of or during admission. For the purpose of this study, a pathogen was defined as a microorganism with the potential to cause disease; the association of the microorganism with a clinically relevant infection was not assessed. Microorganism identification was based on various tests, including conventional cultures, molecular tests, urine antigen tests, and blood serology, as performed by individual local laboratories. *Aspergillus* identification was based on culture data; galactomannan immunoassays were not included. Pathogens were identified from blood, respiratory tract (upper/lower), urine, intra-abdominal, skin/wound, and other sources and classified as Gram-negative bacteria, Gram-positive bacteria, acid fast bacilli, fungi, or viruses. Results likely to be associated with environmental or surveillance specimens (e.g., rectal or nasal swabs) were excluded by use of a previously described algorithmic methodology [10], but nasopharyngeal or nasal swabs used for SARS-CoV-2 testing and also tested for other respiratory pathogens were included. Admission-onset infections were defined as those occurring within 3 days of admission. Pathogens identified outside this period were considered hospital-onset infections. Antimicrobial utilization was defined as an order for ≥24 h of antibiotic therapy at any point during the admission.

The study dataset was approved as a limited, de-identified dataset for retrospective analysis and was exempted from patient consent by the New England Institutional Review Board (Wellesley, Massachusetts) (No. 120180023).

### Outcomes

The primary outcome was the rate of non-SARS-CoV-2 pathogen detection for SARS-COV-2-positive, SARS-CoV-2-negative, and SARS-CoV-2-untested patients. Secondary outcomes included rates of specific pathogens, time period of pathogen isolation (admission onset versus hospital onset), antimicrobial usage, and length of stay (LOS) for the three groups.

### Statistical analysis

Statistical analysis involved descriptive data for observed patients, including pathogens, antibiotic therapy, and LOS. Continuous variables were assessed for normality by use of density and quantile-quantile plots. Univariate comparisons between categorical subgroups were performed using chi-square or Fisher’s exact tests. Continuous variables were analyzed using Kruskal-Wallis tests and Wilcoxon test for pairwise comparisons. All statistical tests were performed with a pre-specified two-tailed alpha level of 0.05. Analyses were conducted using R (R Ver. 4.0.2, R Foundation for Statistical Computing, Vienna, Austria) with RStudio (Boston, MA).

## Results

From March 1 to May 31, 2020, 141,621 of 590,960 patients (24.0%) were tested for SARS-CoV-2 in 241 medical facilities and 449,339 (76.1%) were not tested. A total of 17,003 (12.0%) tested patients were positive for SARS-CoV-2 and 124,618 (88.0%) were negative (Table 1). The proportion of male patients was significantly higher in SARS-CoV-2-positive (53.1%) vs –negative (46.5%) or untested (44.5%) patients. Mean (standard deviation [SD]) age was significantly higher in the SARS-CoV-2-positive group (61.7 [18.0] vs 58.5 [20.9] years for SARS-CoV-2-negative and 48.7 [27.3] years for untested patients). Medical facility characteristics and geographic regions by SARS-CoV-2 status are shown in Supplementary Table 1 (see Additional file 1). Of the 241 facilities, 82.6% were urban hospitals; the bed count was < 100 in 34.9%, 100 to 300 in 39.4%, and > 300 in 25.7%. The census division with the greatest number of facilities was West South Central (25.3%) followed by East North Central (17.4%). At the patient level, the highest SARS-CoV-2-positive rates were in the Northeast (4392/22,364 tested patients [19.6%]) and in hospitals that were urban (16,649/131,726 [12.6%]), teaching hospitals (10,405/78,250 [13.3%]), and with > 300 beds (10,475/79,922 [13.1%]).

**Table 1:** Baseline characteristics and microbiology testing by SARS-CoV-2 and testing status

Characteristic	Untested for SARS-CoV-2 (*n* = 449,339)	Tested for SARS-CoV-2	
		SARS-CoV-2 negative (*n* = 124,618)	SARS-CoV-2 positive (*n* = 17,003)
Demographics			
Male sex, n (%)	199,732 (44.5%)	57,924 (46.5%)	9026 (53.1%)
Age, mean (SD) years	48.7 (27.3)	58.5 (20.9)	61.7 (18.0)
Specimens collected for non-SARS-CoV-2 pathogens^a^ n (%)			
Yes	188,057 (41.9%)	114,550 (91.9%)	16,637 (97.8%)
No	261,282 (58.1%)	10,068 (8.1%)	366 (2.1%)
Specimens positive for non-SARS-CoV-2 pathogens^a^			
Any pathogen, n (% of patients with specimens collected)	52,418 (27.9%)	24,442 (21.3%)	3473 (20.9%)
Multiple pathogens, n (% of specimens with any pathogen)	17,871 (34.1%)	9101 (37.2%)	1192 (34.3%)

^a^Defined as any bacteria, fungus, or virus other than SARS-CoV-2

Most patients tested for SARS-CoV-2 had specimens collected for microbiologic testing for additional pathogens, although the rate was slightly higher for SARS-CoV-2-positive patients (97.8%) compared with SARS-CoV-2-negative patients (91.9%). Among those not tested for SARS-CoV-2, 41.9% of patients had specimens collected for microbiologic testing. The proportion of patients with non-SARS-CoV-2 pathogens detected was highest in patients not tested for SARS-CoV-2 (27.9%) and similar between the SARS-CoV-2-positive (20.9%) and −negative (21.3%) groups (Table 1). The presence of multiple non-SARS-CoV-2 pathogens was slightly more common in SARS-CoV-2-negative patients (37.2% versus 34.3% for SARS-CoV-2-positive and 34.1% for untested patients) (Table 1).

### Specimen sources and pathogens by SARS-CoV-2 and testing status

Because patients could have more than one positive specimen and pathogen, the totals for positive specimens were higher than the number of patients with specimens positive for non-SARS-CoV-2 pathogens (Table 2). About three-quarters of positive specimens in the SARS-CoV-2-negative and −untested groups were collected during the admission period and therefore likely represented community-acquired coinfections. SARS-CoV-2-positive patients had an approximately two-fold higher rate of pathogens detected during the hospital-onset period, which likely represented hospital-acquired infections (42.4% vs 22.2 and 19.5% for SARS-CoV-2-negative and −untested, respectively).

**Table 2:** Onset, source, and non-SARS-CoV-2 pathogens^a^ by SARS-CoV-2 and testing status

Specimen onset, source, and pathogens n (% positive specimens)	Untested for SARS-CoV-2	Tested for SARS-CoV-2		*P* value
		SARS-CoV-2 negative	SARS-CoV-2 positive	
Any positive non-SARS-CoV-2 specimen^b^	73,315 (100%)	38,753 (100%)	5012 (100%)	
**Timing of non-SARS-CoV-2 pathogen detection**				< 0.05
Admission period	59,016 (80.5%)	30,141 (77.8%)	2889 (57.6%)	
Hospital-onset period (> 3 days)	14,299 (19.5%)	8612 (22.2%)	2123 (42.4%)	
**Non-SARS-CoV-2 specimen source**				< 0.05
Urine	20,807 (28.4%)	10,077 (26.0%)	1697 (33.9%)	
Respiratory	9626 (13.1%)	5435 (14.0%)	1228 (24.5%)	
Blood	11,185 (15.3%)	9530 (24.6%)	1020 (20.4%)	
Other	16,393 (22.4%)	7474 (19.3%)	776 (15.5%)	
Skin/Wound	14,033 (19.1%)	5698 (14.7%)	271 (5.3%)	
Intra-abdominal	1271 (1.7%)	539 (1.4%)	20 (0.4%)	
**Non-SARS-CoV-2 pathogens**^**a**^				
Any bacteria	56,215 (76.7%)	30,952 (79.9%)	3972 (79.2%)	
Gram-negative	31, 887 (43.5%)	17,087 (44.1%)	2280 (45.5%)	< 0.05
Enterobacterales	22,289 (30.4%)	11,939 (30.8%)	1594 (31.8%)	0.06
*Pseudomonas aeruginosa*	2729 (3.8%)	1837 (4.7%)	324 (6.5%)	< 0.05
*Stenotrophomonas maltophilia*	302 (0.4%)	238 (0.6%)	39 (0.8%)	< 0.05
*Acinetobacter baumannii*	145 (0.2%)	84 (0.2%)	13 (0.3%)	0.56
Gram-positive	24,035 (32.8%)	13,532 (34.9%)	1670 (33.3%)	< 0.05
*Staphylococcus aureus*	10,147 (13.8%)	6120 (15.8%)	689 (13.7%)	< 0.05
*Enterococcus* spp.	4124 (5.6%)	2357 (6.1%)	357 (7.1%)	< 0.05
*Streptococcus pneumoniae*	682 (0.9%)	513 (1.3%)	94 (1.9%)	< 0.05
Non-SARS-CoV-2 virus	13,150 (17.9)	5403 (13.9%)	543 (10.8%)	< 0.05
Respiratory viruses	4398 (6.0%)	1170 (3.0%)	111 (2.2%)	< 0.05
Fungi	2124 (2.9%)	1328 (3.4%)	280 (5.6%)	< 0.05
*Candida* spp.	2990 (4.1%)	1764 (4.6%)	410 (8.2%)	< 0.05
*Aspergillus* spp.	104 (0.1%)	108 (0.3%)	6 (0.1%)	< 0.05

^a^Any bacteria, fungus, or virus other than SARS-CoV-2

^b^Patients could have more than one positive specimen and pathogen

For all three groups, the most frequent source of positive specimens was urine; urinary pathogens were more common in SARS-CoV-2-positive patients (33.9%) compared with SARS-CoV-2-negative patients (26.0%) or untested patients (28.4%) (Table 2). Respiratory pathogens were identified more frequently in SARS-CoV-2-positive patients (24.5%) compared with SARS-CoV-2-negative (14.0%) and −untested (13.1%) patients. SARS-CoV-2-negative and −untested patients had higher proportions of skin/wound pathogens detected (14.7 and 19.1%, respectively) compared with SARS-CoV-2-positive patients (5.3%).

Bacteria accounted for approximately 80% of pathogens in all three groups (Table 2). Gram-negative bacteria, mostly Enterobacterales, were more common than Gram-positive bacteria, primarily *Staphylococcus aureus* and *Enterococcus* spp. The three groups had generally comparable rates of bacterial pathogens, but significantly higher rates of *Pseudomonas aeruginosa, Stenotrophomonas maltophilia, Enterococcus* spp., and *Streptococcus pneumoniae w*ere observed in the SARS-CoV-2-positive group. Rates of non-SARS-CoV-2 respiratory viruses were significantly lower in the SARS-CoV-2-positive group (Table 2). The proportion of patients with specimens positive for *Candida* spp. was almost twice as high in SARS-CoV-2-positive patients (8.2%) compared with SARS-CoV-2-negative (4.6%) and −untested (4.1%) patients.

### Antimicrobial usage by SARS-CoV-2 and testing status

The rate of antimicrobial usage was significantly higher in SARS-CoV-2-positive (68.0%) versus −negative (45.2%) and −untested (25.1%) patients. The mean (SD) time to the first antibiotic order was similar between the two SARS-CoV-2-tested groups (0.9 [2.6] days for SARS-CoV-2- positive and 0.9 [2.3] days for SARS-CoV-2-negative) but significantly longer in the untested group (1.4 [5.8] days). Mean (SD) duration of antibiotic treatment was 6.1 (5.2) days for SARS-CoV-2-positive patients compared with 4.8 (4.5) for −negative and 4.1 (3.9) for −untested patients.

The most frequently prescribed antimicrobial class in all three groups was cephalosporins, mostly ceftriaxone (Table 3). An order for a macrolide, primarily azithromycin, was far more common in SARS-CoV-2-positive patients (23.4%) compared with SARS-CoV-2-negative (9.6%) and −untested (6.3%) patients.

**Table 3:** Antimicrobial usage by SARS-COV-2 and testing status

Antimicrobial n (% of total antimicrobials)	Not tested for SARS-CoV-2 (*n* = 452,024)	Tested for SARS-CoV-2	
		SARS-CoV-2 negative (*n* = 124,927)	SARS-CoV-2 positive (*n* = 17,049)
Total number of antimicrobials^a^	225,692 (100%)	133,735 (100%)	31,981 (100%)
Antibacterial	211,088 (93.5%)	125,811 (94.1%)	29,828 (93.3%)
Cephalosporin	61,468 (27.2%)	37,514 (28.1%)	9142 (28.6%)
Ceftriaxone	32,198 (14.3%)	20,081 (15.0%)	5864 (18.3%)
Cefepime	13,201 (5.8%)	10,463 (7.8%)	2647 (8.3%)
Macrolides	14,200 (6.3%)	12,883 (9.6%)	7482 (23.4%)
Azithromycin	13,632 (6.0%)	12,642 (9.5%)	7461 (23.4%)
Glycopeptides	37,961 (16.8%)	23,586 (17.6%)	4254 (13.3%)
Vancomycin	37,955 (16.8%)	23,585 (17.6%)	4254 (13.3%)
Beta-lactam/beta-lactamase inhibitors	31,642 (14.0%)	17,997 (13.5%)	3065 (9.6%)
Piperacillin/tazobactam	24,729 (11.0%)	14,145 (10.6%)	1981 (6.2%)
Tetracyclines	6668 (3.0%)	5585 (4.2%)	2038 (6.4%)
Carbapenems	7684 (3.4%)	5068 (3.8%)	1145 (3.6%)
Fluoroquinolones	14,171 (6.3%)	7119 (5.3%)	800 (2.5%)
Antivirals	8055 (3.6%)	3918 (2.9%)	1574 (4.9%)
Neuraminidase inhibitors	2748 (1.2%)	823 (0.6%)	950 (3.0%)
Antifungals	6549 (2.9%)	4006 (3.0%)	579 (1.8%)

^a^Patients could have more than one prescribed antibiotic

### Outcomes by SARS-CoV-2 and testing status

Hospital and ICU LOS were significantly longer for SARS-CoV-2-positive versus SARS-CoV-2-negative and −untested patients (Table 4). A positive pathogen specimen was associated with a longer LOS in all groups. In the SARS-CoV-2-positive group, the mean (SD) length of stay with a positive pathogen specimen was 13.7 [15.7] days vs 8.2 (11.5) days for patients with a negative pathogen specimen. LOS was further increased by the presence of multiple pathogens (17.5 [16.1] days for SARS-CoV-2-positive patients).

**Table 4:** Length of stay by SARS-CoV-2, testing, and non-SARS-CoV-2 pathogen status

Admission site and pathogen status	Not tested for SARS-CoV-2		Tested for SARS-CoV-2			
			SARS-CoV-2 negative		SARS-CoV-2 positive	
	n	LOS	n	LOS	n	LOS
Hospital^a^	449,339	4.2 (8.0) [2]	124,618	5.1 (8.9) [3]	17,003	8.6 (11.4) [6]
Positive for non-SARS-CoV-2 pathogen	52,418	7.1 (11.0) [4]	24,442	8.2 (11.5) [5]	3483	13.7 (15.7) [9]
Negative for non-SARS-CoV-2 pathogen	396,921	3.9 (7.4) [2]	100,176	4.3 (7.9) [3]	13,566	7.3 (9.6) [5]
Multiple non-SARS-CoV-2 pathogens	17,871	8.7 (13.2) [5]	9101	10.2 (14.6) [6]	1192	17.5 (16.1) [14]
ICU^a^	47,629	3.6 (5.9) [2]	21,060	3.8 (6.2) [2]	4076	7.8 (8.5) [5]

Data are expressed as mean (standard deviation) [median] days. *P* < 0.05 across groups for all outcomes

Note: *ICU* intensive care unit, *LOS* length of stay

^a^Overall hospital and ICU LOS include patients with no specimen collected

## Discussion

Experts have identified an urgent need for accurate information on potential coinfection and superinfection rates in patients with SARS-CoV-2 to help inform diagnosis, disease management, and antimicrobial stewardship during the COVID-19 pandemic [3–6]. In our study, there was a high rate of testing for non-SARS-CoV-2 pathogens among those tested for SARS-CoV-2 infection; approximately 20% of patients tested for SARS-CoV-2 had a positive specimen for an additional pathogen, regardless of SARS-CoV-2 status. However, SARS-CoV-2-positive patients had higher rates of hospital-onset pathogens, greater antimicrobial usage, and extended hospital and ICU LOS compared with SARS-CoV-2-negative or −untested patients.

During our 3-month study (March to May), 12% of hospitalized adult patients tested for SARS-CoV-2 in US acute care facilities were positive for this virus. As might be expected, testing for additional pathogens was far more common in patients tested for SARS-CoV-2 (more than 90%) compared with patients that were not tested for SARS-CoV-2 (42%), as many untested patients were likely admitted for noninfectious causes with no reason to expect or test for a clinically relevant pathogen. Nevertheless, the rate of pathogen detection was slightly higher among untested patients (28% versus 21%). Urine was the most frequent specimen source for pathogens in all groups, followed by respiratory samples for SARS-CoV-2-positive patients. The relatively low yield of non-SARS-CoV-2 pathogens across all subgroups may be relevant to hospitals coping with the redeployment of diagnostics to focus on the COVID-19 pandemic [4, 11].

In presenting these data, it is important to note that we do not consider the SARS-CoV-2-negative or −untested subgroups to be “controls” for the SARS-CoV-2-positive patients in this study, as the subgroups differed in many important characteristics including the proportion of patients with hospital-onset pathogens. Rather, we believe the inclusion of these other subgroups provides context for the findings in the SARS-CoV-2 tested subgroup.

Other studies have come to varying conclusions concerning rates of coinfection in patients with SARS-CoV-2 [11–15]. The differences among studies may relate to study location and to testing procedures. The strength of our study is use of a broader approach on two levels: 1) we assessed all specimens collected during the admission, rather than focusing solely on respiratory pathogens; and 2) we included regionally diverse hospitals throughout the US. Given the extensive geographic variations in SARS-CoV-2 infection rates during the course of the pandemic [16, 17] and region-specific patient characteristics [18], national data may provide a more complete view of SARS-CoV-2 infections and attendant coinfections.

Rates of specific pathogens were generally comparable among the three groups, although rates of some bacteria, including *P. aeruginosa*, and rates of *Candida* spp. were significantly higher in SARS-CoV-2-positive patients compared with −negative or −untested patients. Both pathogens are frequently found in the respiratory tract of ventilated patients or critically ill patients, and both are associated with previous antibiotic treatment [19]. In contrast, rates of *Aspergillus* were highest in SARS-CoV-2-negative patients. Variability in methods used to detect *Aspergillus* impact reported rates of infection [20]. Studies using various methods have reported pulmonary aspergillosis rates of 20 to 30% in the most severely ill, mechanically ventilated COVID-19 patients [20], but the overall rate is much lower. In line with the numbers reported here, a recent study identified COVID-19-associated pulmonary aspergillosis in 0.3% of all hospitalized COVID-19 patients [21].

In SARS-CoV-2-positive patients, 58% of non-SARS-CoV-2 pathogens were identified in specimens collected within 3 days of admission. The delayed onset of the remaining 42% of positive pathogen detections indicates the increased risk of healthcare exposure for the acquisition of opportunistic pathogens and/or healthcare-associated infections. In contrast, positive pathogen detection in specimens collected within 3 days of admission occurred at higher rates in SARS-CoV-2-negative (78%) and −untested (81%) patients, and rates of subsequent hospital-onset positive pathogen detections were correspondingly reduced. The different temporal distribution of positive specimens among these three groups suggests that characteristics associated with hospitalized COVID-19 patients [3], including comorbidities, the severity of illness associated with SARS-CoV-2, extended hospital lengths of stay, and the frequent need for ventilation [15, 22], perhaps in conjunction with immunomodulatory treatments, may increase susceptibility to infection with other pathogens [3]. In addition, prolonged SARS-CoV-2 infection could itself potentially confer an immunosuppressed state that increases vulnerability to opportunistic infections [23]. Rates of healthcare-associated infections may have also been impacted by the heavy burden on hospitals during COVID-19 surges, leading US medical professional societies to urge the Department of Health and Human Services to suspend reimbursement penalties for healthcare-associated infections during the pandemic [24]. Given the fact that urine and respiratory sources predominated as sources of pathogens in SARS-CoV-2-positive patients, it is feasible that infection prevention bundles and the use of personal protective equipment may have been adversely affected as a result of the pandemic [25, 26].

Histopathologic studies support an important role for *S. pneumoniae* and other Gram-positive pathogens, including *Streptococcus pyogenes* and *S. aureus*, in contributing to lung damage and causing bacterial pneumonia in patients with influenza [27]. Gram-negative pathogens have been identified as the major cause of bacterial pneumonia in critically ill patients with COVID-19 [28]. In addition to lung damage, pathogens can influence a number of other pathologic processes. For instance, during the influenza A H1N1 pandemic in 2009, coinfection with *S. aureus* was reported to be associated with an increased risk of severe coagulopathy in children [29]. It should be noted, however, that the correlation between copathogens and other severe clinical outcomes is not necessarily causative; the presence of copathogens might instead act as a marker for more severe chronic conditions or reflect differences in individual immune responses.

In our study, SARS-CoV-2-positive patients had longer hospital and ICU LOS compared with SARS-CoV-2-negative or −untested patients. Identification of a pathogen was associated with increased LOS in all groups, but was particularly notable in SARS-CoV-2-positive patients, who had a mean hospital LOS of almost 14 days in the SARS-CoV-2-positive/pathogen specimen-positive group compared with 7 days in the SARS-CoV-2-positive/pathogen specimen-negative group. The presence of multiple pathogens further increased LOS. These data are derived from patients with additional pathogens identified during both the admission onset and hospital onset periods.

Antimicrobial usage was significantly higher in SARS-CoV-2-positive patients (68% vs 45% for SARS-CoV-2-negative and 25% for −untested patients), despite similar or lower rates of pathogen-positive specimens. The higher rate of antimicrobial use may be due to initiation of empiric therapy for suspected pneumonia or sepsis, consistent with guidelines for the latter [30]. Other studies have also observed higher utilization of antibiotics in patients with SARS-CoV-2 with no identified coinfection [4, 31, 32]. Although most drug classes were used at comparable levels in the different groups, the use of macrolides was much higher in SARS-CoV-2-positive patients. This may be due to the widely reported increase in radiographic opacities in COVID-19 patients concurrent with signs and symptoms of pneumonia at presentation [33]. Macrolide use was also likely influenced by early reports concerning the potential efficacy of hydroxychloroquine plus azithromycin in managing COVID-19 [34], which were not supported by subsequent studies [35, 36].

The treatment patterns observed in our study likely reflect difficulties in making treatment decisions in critically ill patients, prolonged turnaround time for SARS-CoV-2 testing, particularly in the early days of the pandemic [37], and concerns over potential bacterial superinfection in SARS-CoV-2, but may also indicate overuse of antimicrobials. Collateral damage from antimicrobial overuse includes increased selection of antimicrobial resistance and opportunistic pathogens such as *C. difficile*, adverse effects of drugs, and unnecessary treatment costs [3, 4, 9, 38]. Continued monitoring of the utilization and appropriateness of antimicrobial use among SARS-CoV-2 patients is required to identify antimicrobial stewardship opportunities.

As with any database study, the study reported here has important limitations. SARS-CoV-2 and pathogen status were based on reports from institutional facilities; there was no uniform method of testing or central laboratory. The sensitivity and specificity of SARS-CoV-2 PCR assays are known to vary based on several factors, including time from exposure [39, 40]. No case definition for COVID-19 disease was applied, consistent with current medical care practices, and we did not assess disease severity, so it is possible that some of the SARS-CoV-2 patients identified here were asymptomatic and admitted for other causes. Although our established algorithm [10] is designed to remove admissions with colonizing microbes from the analyses, some of the pathogens resulting in positive specimens may not have been associated with clinically significant infections. Certain geographic areas may have been underrepresented by our database, although the northeast, which had high SARS-CoV-2 rates in the earliest phase, is well represented. Influenza is not common during the months included in this study, so our findings concerning co-infection with respiratory viruses may not be representative of other times of the year. Our study reflects an early period in the COVID-19 pandemic; it will be important to follow changes in these patterns over time to assess changes in virus epidemiology and in healthcare resource utilization.

The findings reported in our study raise several important points of clinical relevance. First, potential copathogens (concurrent infections or superinfections) occur in approximately 21% of SARS-CoV-2 patients, so the identification of another pathogen does not rule out the presence of SARS-CoV-2. Second, although overall potential copathogen rates were similar regardless of SARS-CoV-2 status, clinicians should be aware of the increased risk of secondary infections in patients with SARS-CoV-2 and the burden associated with copathogens. The presence of an additional pathogen was associated with a marked increase in hospital and ICU LOS for all groups, but the difference was greatest in SARS-CoV-2-positive patients. Third, about 80% of SARS-CoV-2-positive patients do not have a detectable copathogen, so antimicrobial therapy should be evaluated daily and de-escalated when possible. Our data underscore the need for sensitive and specific diagnostics to help inform appropriate antimicrobial stewardship. The deployment of early respiratory diagnostic tests relative to the first administration of an antimicrobial is therefore a prime consideration, especially for septic patients who will likely receive an antimicrobial within the first hour of acute care.

During the 3-month period encompassed by this study, SARS-CoV-2-positive patients had specimens collected more than twice as often as patients not tested for SARS-CoV-2. Additionally, SARS-CoV-2-positive patients were 1.5-fold more likely to be prescribed antibiotics than SARS-CoV-2-negative patients, and 2.7-fold more likely than untested patients. Our findings document epidemiological variations in specimen collection, pathogen prevalence, and antimicrobial utilization associated with SARS-CoV-2 status that may be useful in helping clinicians assess and manage patients with suspected or confirmed COVID-19, and highlight the considerable operational burden that COVID-19 imposes on healthcare systems nationwide.

## Supplementary Information

**Additional file 1: Supplementary Table 1.** Patients by Medical Facility Characteristics and Geographic Location.

## Data Availability

The datasets used and/or analysed during the current study are available from the corresponding author on reasonable request.
