# Supplementary material for: A multicenter analysis of the clinical microbiology and antimicrobial usage in hospitalized patients in the US with or without COVID-19
Source: BMC Infect Dis. 2021 Feb 27;21:227. doi: 10.1186/s12879-021-05877-3 (PMC7910773; doi:10.1186/s12879-021-05877-3)
Supplement: Supplementary file 1 — Additional file 1: Supplementary Table 1. Patients by Medical Facility Characteristics and Geographic Location. [file 12879_2021_5877_MOESM1_ESM.docx]

**Supplementary Information**

**A multicenter analysis of the clinical microbiology and antimicrobial usage in hospitalized patients in the US with or without COVID-19**

Laura Puzniak^1*^, Lyn Finelli^1^, Kalvin C. Yu^2^, Karri A. Bauer^1^, Pamela Moise^1^ Carisa De Anda^1^, Latha Vankeepuram^2^, Aryana Sepassi^2^, Vikas Gupta^2^

^1^Merck & Co., Inc., Kenilworth, NJ, USA

^2^Becton, Dickinson and Company, Franklin Lakes, NJ, USA

**Supplementary Table 1**. Facility Distribution and Patients by Medical Facility Characteristics and Geographic Location

| **Characteristic** | **Facilities**  **(n= 241)** | **Patients** | | |
| --- | --- | --- | --- | --- |
|  |  | **Not tested for  SARS-CoV-2**  **(n=449,339)** | **Tested for SARS-CoV-2** | |
|  |  |  | **SARS-CoV-2 negative**  **(n=124,618)** | **SARS-CoV-2 positive**  **(n=17,003)** |
| Metropolitan status |  |  |  |  |
| Urban | 199 (82.6%) | 402,314 (89.5%) | 115,077 (92.3%) | 16,649 (97.9%) |
| Rural | 42 (17.4%) | 47,025 (10.5%) | 9,541 (7.7%) | 354 (2.1%) |
| Bed count |  |  |  |  |
| <100 | 84 (34.9%) | 41,292 (9.2%) | 9,049 (7.3%) | 741 (4.4%) |
| 100 to 300 | 95 (39.4%) | 164,464 (36.6%) | 46,122 (37.0%) | 5,787 (34.0%) |
| >300 | 62 (25.7%) | 243,583 (54.2%) | 69,447 (55.7%) | 10,475 (61.6%) |
| Facility type |  |  |  |  |
| Teaching | 61 (25.3%) | 206,460 (45.9%) | 67,845 (54.4%) | 10,405 (61.2%) |
| Non-teaching | 156 (64.7%) | 231,320 (51.5%) | 54,282 (43.6%) | 6,568 (38.6%) |
| Specialty | 19 (7.9%) | 5,195 (1.2%) | 1,311 (1.1%) | 19 (0.1%) |
| Children’s | 4 (1.7%) | 6,364 (1.4%) | 1,180 (0.9%) | 11 (0.1%) |
| Medical school affiliation |  |  |  |  |
| Graduate | 12 (5.0%) | 31,589 (7.0%) | 9,562 (7.7%) | 1,124 (6.6%) |
| Limited | 30 (12.4%) | 116,318 (25.9%) | 31,237 (25.1%) | 3,606 (21.2%) |
| Major | 24 (10.0%) | 74,955 (16.7%) | 25,852 (20.7%) | 5,218 (30.7%) |
| No affiliation | 175 (72.6%) | 226,477 (50.4%) | 57,967 (46.5%) | 7,055 (41.5%) |
| CDC region |  |  |  |  |
| Midwest | 46 (19.1%) | 83,561 (18.6%) | 26,575 (21.3%) | 5,091 (29.9%) |
| Northeast | 31 (12.9%) | 58,253 (13.0%) | 17,972 (14.4%) | 4,392 (25.8%) |
| South | 127 (52.7%) | 246,687 (54.9%) | 65,908 (52.9%) | 6,292 (37.0%) |
| West | 37 (15.4%) | 60,838 (13.5%) | 14,163 (11.4%) | 1,228 (7.3%) |
| Census division |  |  |  |  |
| East North Central | 42 (17.4%) | 75,356 (16.8%) | 23,969 (19.2%) | 4,856 (28.5%) |
| East South Central | 36 (14.9%) | 59,118 (13.2%) | 17,911 (14.4%) | 1,343 (7.9%) |
| Mid-Atlantic | 26 (10.8%) | 49,939 (11.1%) | 14,245 (11.4%) | 3,549 (20.9%) |
| Mountain | 11 (4.6%) | 13,134 (2.9%) | 3,592 (2.9%) | 151 (0.9%) |
| New England | 5 (2.1%) | 8,314 (1.9%) | 3,727 (3.0%) | 843 (5.0%) |
| Pacific | 16 (6.6%) | 47,704 (10.6%) | 10,571 (8.4%) | 1,077 (6.3%) |
| South Atlantic | 30 (12.4%) | 96,584 (21.5%) | 25,360 (20.4%) | 3,056 (18.0%) |
| West North Central | 14 (5.8%) | 8,205 (1.8%) | 2,606 (2.1%) | 235 (1.4%) |
| West South Central | 61 (25.3%) | 90,985 (20.2%) | 22,637 (18.2%) | 1,893 (11.1%) |

Data are presented as number (%) of facilities and number of patients (%).
